# Natural durability of timber species exposed to xylophagous fungi in southern Durango, Mexico

**DOI:** 10.7717/peerj.14541

**Published:** 2023-03-10

**Authors:** Yolanda Ontiveros-Moreno, Serafín Colín-Urieta, José Javier Corral-Rivas, José Ciro Hernández-Díaz, José Ángel Prieto-Ruíz, Artemio Carrillo-Parra

**Affiliations:** 1 Programa Institucional de Doctorado en Ciencias Agropecuarias y Forestales (PIDCAF), Universidad Juárez del Estado de Durango (UJED), Durango, Durango, México; 2 Facultad de Ingeniería en Tecnología de la Madera, Universidad Michoacana de San Nicolás de Hidalgo, Morelia, Michoacán, México; 3 Facultad de Ciencias Forestales y Ambientales (FCFyA), Universidad Juárez del Estado de Durango, Durango, Durango, México; 4 Instituto de Silvicultura e Industria de la Madera (ISIMA), Universidad Juárez del Estado de Durango (UJED), Durango, Durango, Mexico

**Keywords:** *Coniophora puteana*, *Trametes versicolor*, *Pinus* spp, *Juniperus deppeana*, Natural durability, *Xylophagus fungi*

## Abstract

**Introduction:**

Wood is a natural resource used for construction and the manufacture of many products. This material is exposed to damage due to biotic and abiotic factors. An important biotic factor is wood-degrading fungi that generate large economic losses. The objectives of this study were to determine the effect of xylophagous fungi (*Coniophora puteana* and *Trametes versicolor*) on the natural durability of six timber species in southern Durango, Mexico, and to establish differences between fungal effects on each tree species.

**Materials and Methods:**

Samples of *Pinus durangensis, P. cooperi, P. strobiformis, Juniperus deppeana, Quercus sideroxyla*, and *Alnus acuminata* were exposed to fungi for 4 months under laboratory conditions according to European Standard EN350-1. Samples of *Fagus sylvatica* were used as control. Durability was determined as the percentage of wood mass loss for each species. Welch ANOVA tests were performed to establish differences among tree species. Welch t-tests were used to prove loss mass differences between fungi for each tree species.

**Results:**

The most resistant species to *C. puteana* were *P. durangensis, J. deppeana*, *P. cooperi* and *P. strobiformis*, showing mean mass losses lower than 8.08%. The most resistant species to *T. versicolor* were *J. deppeana*, *P. strobiformis* and *P. durangensis* (mean mass losses lower than 7.39%). *Pinus strobiformis* and *Q. sideroxyla* were more susceptible to *C. puteana* effect; in contrast, *P. durangensis* and *P. cooperi* showed more damage due to *T. versicolor* degradation.

**Conclusions:**

Woods of *P. durangensis, P. cooperi, P. strobiformis* and *Juniperus deppeana* are well adapted to infection by these xylophagous fungi and are therefore highly recommended for commercial use in southern Durango, Mexico.

## Introduction

Wood is a renewable natural forest product that has high economic importance as it serves as a material for construction and the manufacture of a large number of objects (Vázquez, 2014). Durability is the resistance of wood to degradation by abiotic and biotic factors and depends on the tree species and the care that is given to the wood (Colín-Urieta et al., 2015). Abiotic factors such as changes in temperature, high humidity, aerial erosion, rain, and ultraviolet rays cause photodegradation, swelling, cracks, and discoloring of the wood (Trevisan et al., 2008; Torres, Figueroa & Vives, 2011; Hibbett et al., 2016). Biotic factors also affect wood quality.

With respect to biotic factors, fungi are among those that most deteriorate commercial wood (Miklósiová, 2014; Sundararaj et al., 2015); these microorganisms are capable of degrading wood cell walls at different levels by using their components. The main groups of degrading fungi are called white, brown, and soft fungi due to the effect produced on wood. They generate rot in the wood by using chemical substances at different rates, thus producing different patterns of decomposition (Kubicek, 2012). Airborne spores are the main source of rot fungi infection in above ground conditions (Zabel & Morrell, 2012). After prolonged moisture exposure, spores can trigger an infection in unprotected wood (Deacon, 1997). The fungi able to grow in sapwood and heartwood are different; this indicates the influence that different levels of moisture absorption, intermittent warming, condensation processes, or naturally occurring antifungal substances may have. Species that can tolerate the concentration of tannins or other polyphenols can become established in the heartwood (Råberg et al., 2005).

Broadly speaking, fungal development in wood is favored by conducive humidity (60%), pH (alkaline), and temperature (20 °C) levels, as well as the amount of starch and sugars in the cell walls; then fungi establish in plant tissues and feed on organic substances. Wood degradation due to fungi is observed as a dark stain on the sapwood that causes a decrease in quality, thereby decreasing the market price (Zabel & Morrell, 2012; Peraza-Sánchez, 2002). Fungal enzymes weaken the wood by breaking down cellulose and lignin in the cell walls; wood damage depends on the fungal species and the type of compound that each one degrades (Cruz de León, 2010).

Due to their importance as wood destroyers and their wide distribution, some fungal species have been used as a test fungus for wood preservatives in European trials for over 70 years (Kauserud et al., 2007). In this regard, *Coniophora puteana* is a very common threat to wooden buildings and other structures where softwood is used (Hiltunen et al., 2020); the damage it causes has been evidenced more frequently indoors in the so-called subtropical climates of southern European countries (Bogusław Adam, Krajewski & Betlej, 2021). This species is distributed in different forms and in various types of dead wood throughout the world. According to Ramsbottom’s research (Ramsbottom, 1937), a group of fungi, including *C. puteana*, caused enormous rot problems on sailing ships during the 18th century which may have led to the dispersal of these fungi around the world (Kauserud et al., 2007). These fungi produce brown rot, which mainly breaks down cellulose and hemicellulose in wood and leaves modified lignin as a residue, and it can cause a loss of wood mass of up to 70% reducing mechanical resistance and causing dark staining in wood (Hiltunen et al., 2020; Brischke et al., 2013; Hyde & Wood, 1997). On the other hand, according to Karim et al. (2017), *Trametes versicolor* causes more than 90% of white-rotting damage to trees and wood in northern Iran and North America (Bari et al., 2016).

This type of fungi degrades lignin, which affects the strength and compression of the wood; this degradation is called white rot because of its fibrous or even mealy appearance (Luley, 2005).

In Durango, Mexico, there are more than 20 species of timber trees, among which are the genera *Pinus* spp*., Quercus* spp., *Juniperus* spp. and *Alnus* spp. (García & González, 2003). They are widely distributed across the Sierra Madre Occidental and have high economic importance (SEMARNAT, 2010). The wood of these species is exported in the form of sawnwood, venner sheets, plywood, particle boards, OSB boards and MDF boards mainly to the United States and Canada, European Union countries such as Germany, Spain and the United Kingdom, and Asian countries such as Japan, South Korea and China (FAOSTAT, 2020; Torres-Rojo, 2021; Yuan & Tsigas, 2018; Velarde-Moreno, Alarcón-Osuna & Blanco-Jimenez, 2019). However, to date there is little information on the natural durability of Mexican timber species to xylophagous fungal infections. By increasing technological knowledge and their natural durability, the use of these species could be expanded. Due to the above, the objectives of this study were to determine the effect of xylophagous fungi (*Coniophora puteana* and *Trametes versicolor*) on the natural durability of six timber species (*Pinus cooperi*, *P. strobiformis*, *P. durangensis*, *Alnus acuminata*, *Juniperus deppeana* and *Quercus sideroxyla*) in southern Durango, Mexico, and to evaluate differences between fungal effects for each tree species. Samples of *Fagus sylvatica* were used as the control. This information is expected to increase the knowledge needed to support forest management measures that help prevent economic losses related to fungal degradation in Durango, Mexico.

## Materials and Methods

### Collection site

**Figure 1:**
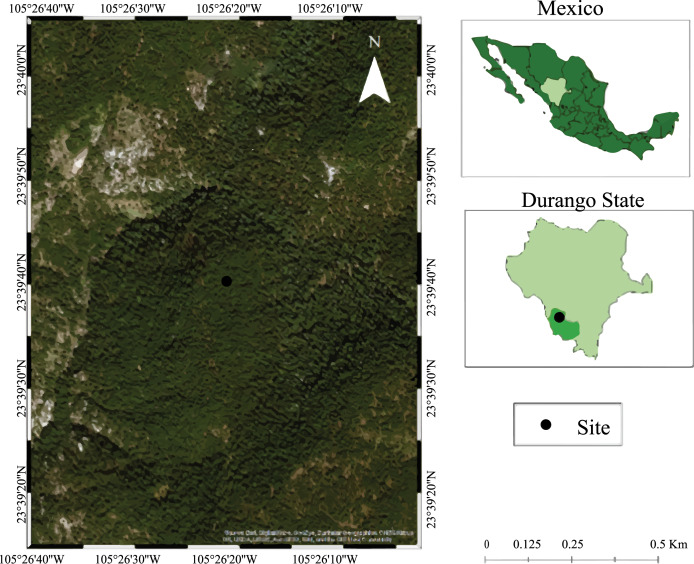
Collection site, Ejido Brillante, municipality of Pueblo Nuevo, Durango, Mexico, Durango-Mazatlan highway, kilometer 99. Figure shows the site where samples were collected in the Ejido Brillante, municipality of Pueblo Nuevo, Durango, Mexico, Durango-Mazatlan highway, kilometer 99.

Wood samples from *Pinus cooperi, P. strobiformis, P. durangensis, Alnus acuminata*, *Juniperus deppeana*, and *Quercus sideroxyla* were collected in the Ejido Brillante, municipality of Pueblo Nuevo, Durango, Mexico (23°39′39″ N and 105°26′20″ W; Fig. 1) in January 2018. *Fagus sylvatica* samples (control) were acquired in a lumber yard in the city of Durango, Mexico (Fig. 1).

### Sawing and dimensioning of specimens

For each tree species, logs (one meter in length and 30 cm in diameter) were first collected and then immediately sawn to make boards, whose dimensions varied depending on the species. Boards were air dried for a month, under temperatures of 1 °C to 20 °C and relative humidity of 64%. A total of 420 samples (3 cm long, 1 cm wide and 0.5 cm thick) were obtained from all the boards, corresponding to 60 samples (30 for each of the two fungal species) for each of the seven timber species. The rings were considered to have an angle between 30° and 70° according to European Standard 113-1 (CEN EN 113, 1996).

### Wooden sample drying and conditioning

Samples were dried in a stove at 103 °C until constant weight was reached; then dry mass was determined on an analytical balance to the nearest 0.01 g. Finally, the samples were conditioned to 20 °C and 65% relative humidity to achieve a moisture content of 12% as is normally found in wood in service.

### Evaluated treatments

Petri dishes containing 0.50 mL of malt agar were prepared. A one cm inoculum of *Coniophora puteana* was placed in each of three Petri dishes. Five wooden samples of *Pinus cooperi* and one of *Fagus sylvatica* were placed around the inoculum at an equal distance of separation. Another three Petri dishes were prepared placing the six wooden samples but without inoculum, serving as controls (leaching). The same was done for each of the seven wood species (a total of 42 Petri dishes). Subsequently, this same experimental design was applied using *Trametes versicolor* as inoculum. For 4 months the Petri dishes were kept under controlled conditions in an incubator (23 °C and 65% relative humidity). Each month the Petri dishes were monitored to avoid contamination from other fungal species.

### Quantification of mass loss

At the end of the growth period of the fungus, the anhydrous weight of the wooden samples of all tree species was determined. Mass loss was estimated using the following formula:

$$P_m=\frac{P_i-P_f}{P_i}\times 100$$

where: Pm = Mass loss (%); Pi = Anhydrous mass of the specimen at the beginning of the test (g); Pf = Anhydrous mass of the specimen at the end of the test (g).

### Classification of natural durability

The natural durability of each species was expressed according to the “X value”, which was obtained by dividing the average mass loss of each species by the mass loss of *F. sylvatica*, and then classified according to EN 350-1 mass loss criteria, where: X values f 0.15 is class 1 = Very durable; 0.15 < X values f 0.30 is class 2 = Durable; 0.30 < X values f 0.60 is class 3 = Moderately durable; 0.60 < X values f 0.90 is class 4 = Slightly durable and X values > 0.90 is class 5 = Not durable (CEN EN 350-1, 1994).

### Statistical analysis

Descriptive statistics of mass loss for each tree species were obtained. Shapiro–Wilk normality and Levene homogeneity of variance tests were performed. Data were normalized by transforming to log10+1 (Zar, 1999). Since the data showed heterogeneity of variance, Welch ANOVA tests were used to observe differences of mass loss among tree species; Games–Howell tests were used to separate groups. Also, Welch t-tests were used to prove mass loss differences between fungi for each tree species. All tests were considered significant at *P* < 0.05 and were conducted in SPSS Ver.18.

## Results

### Durability of wood exposed to *Coniophora puteana*

**Figure 2:**
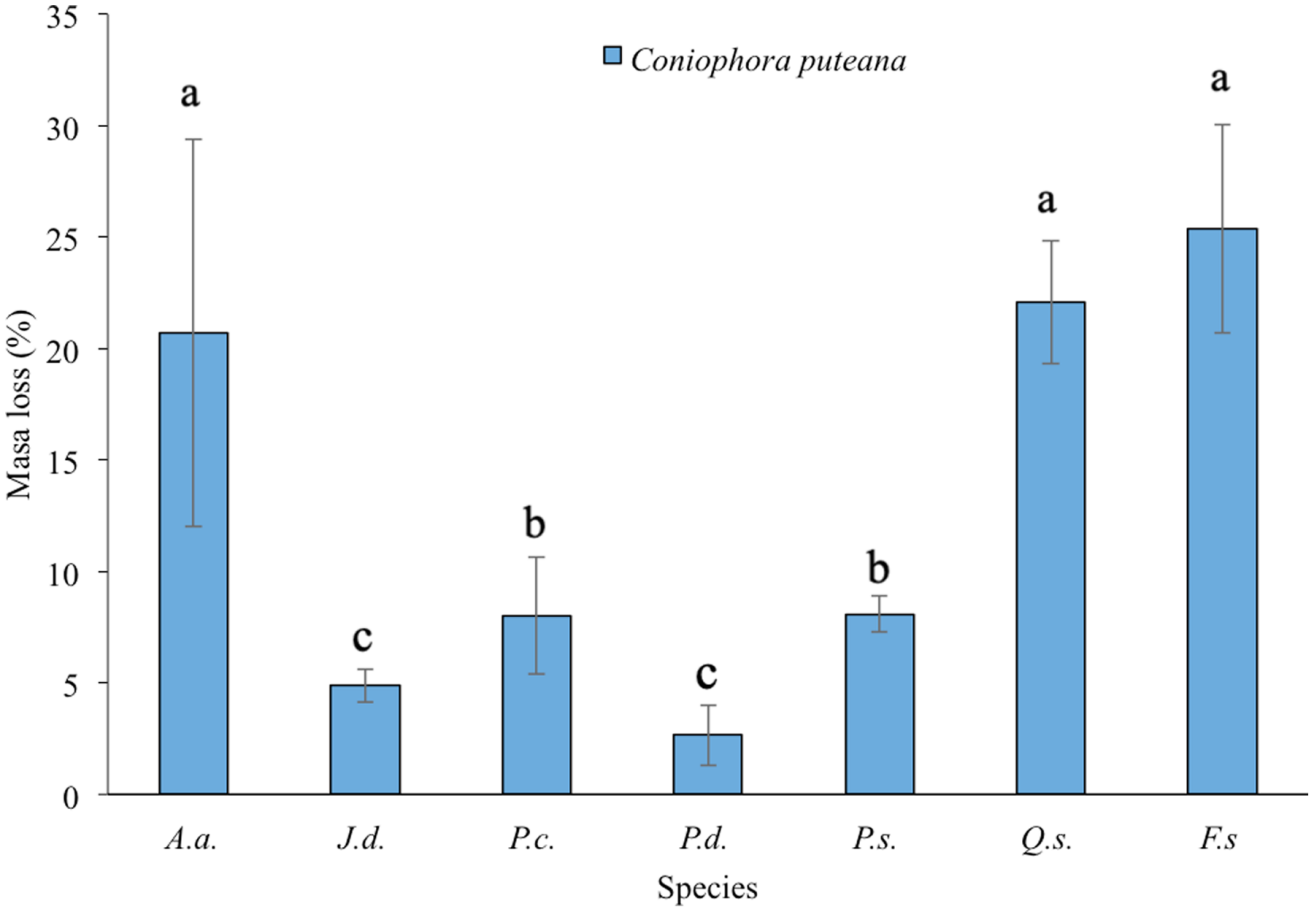
Mean ± SD of mass loss (%) of seven tree species exposed to *Coniophora puteana* in in southern Durango, Mexico. Lowercase letters indicate the significant groups according to the Games–Howell test (*P* < 0.05). *P.d*. = *Pinus durangensis*, *J.d*. = J. The figure shows the mean ± SD of mass loss (%) of seven tree species exposed to *Coniophora puteana* in southern Durango, Mexico.

Significant differences in mass loss were observed among the tree species exposed to *C. puteana* (Welch = 240.78, df = 6, 43.10; *P* < 0.0001; Table 1, Fig. 2). Although this fungal species is considered to deteriorate softwood speces to a greater extent, in this study the highest mass losses were in *Alnus acuminata*, *Q. sideroxyla* and the control *F. sylvatica*, which are in the same statistical group. On the contrary, *Pinus durangensis* showed the lowest mass loss (*x* = 2.65%), followed by *J. deppeana* (*x* = 4.88%). *Pinus cooperi* and *P. strobiformis*, for their part, formed their own statistical group, both losing 8% of their mass.

**Table 1:** Descriptive statistics of mass loss (%) of the seven species of tress exposed to *Coniophora puteana* in southern Durango, Mexico. The values displayed in this table are the mass loss of wood samples of tree species exposed to the fungus *Coniophora puteana*.

Species	*n*	Mean	SE	SD	Min	Max	X value	Durability class
*Alnus acuminata*	15	20.68	2.24	8.68	5.4	33.72	0.82	4
*Juniperus deppeana*	15	4.88	0.19	0.74	3.64	6.07	0.19	3
*Pinus cooperi*	15	8.03	0.68	2.63	4.43	13.28	0.32	3
*Pinus durangensis*	15	2.65	0.35	1.36	1.35	5.21	0.10	1
*Pinus strobiformis*	15	8.08	0.21	0.82	6.42	9.08	0.32	3
*Quercus sideroxyla*	15	22.06	0.71	2.75	17.16	26.55	0.87	5
*Fagus sylvatica*	16	25.36	1.17	4.68	18.16	33.67	1.00	N/A

**Notes:**

X value: calculated from the average mass loss of each wood species divided by the average mass loss of *F. sylvatica* after 16 weeks of fungal incubation.

Durability class: classification system according to mass loss criteria and EN 350-1. 1 = Very durable, 2 = Durable, 3 = Moderately durable, 4 = Slightly durable and 5 = Not durable.

N/A, Not available.

*n*, number of samples; SE, standard error; SD, standard deviation.

### Durability of wood exposed to *Trametes versicolor*

**Figure 3:**
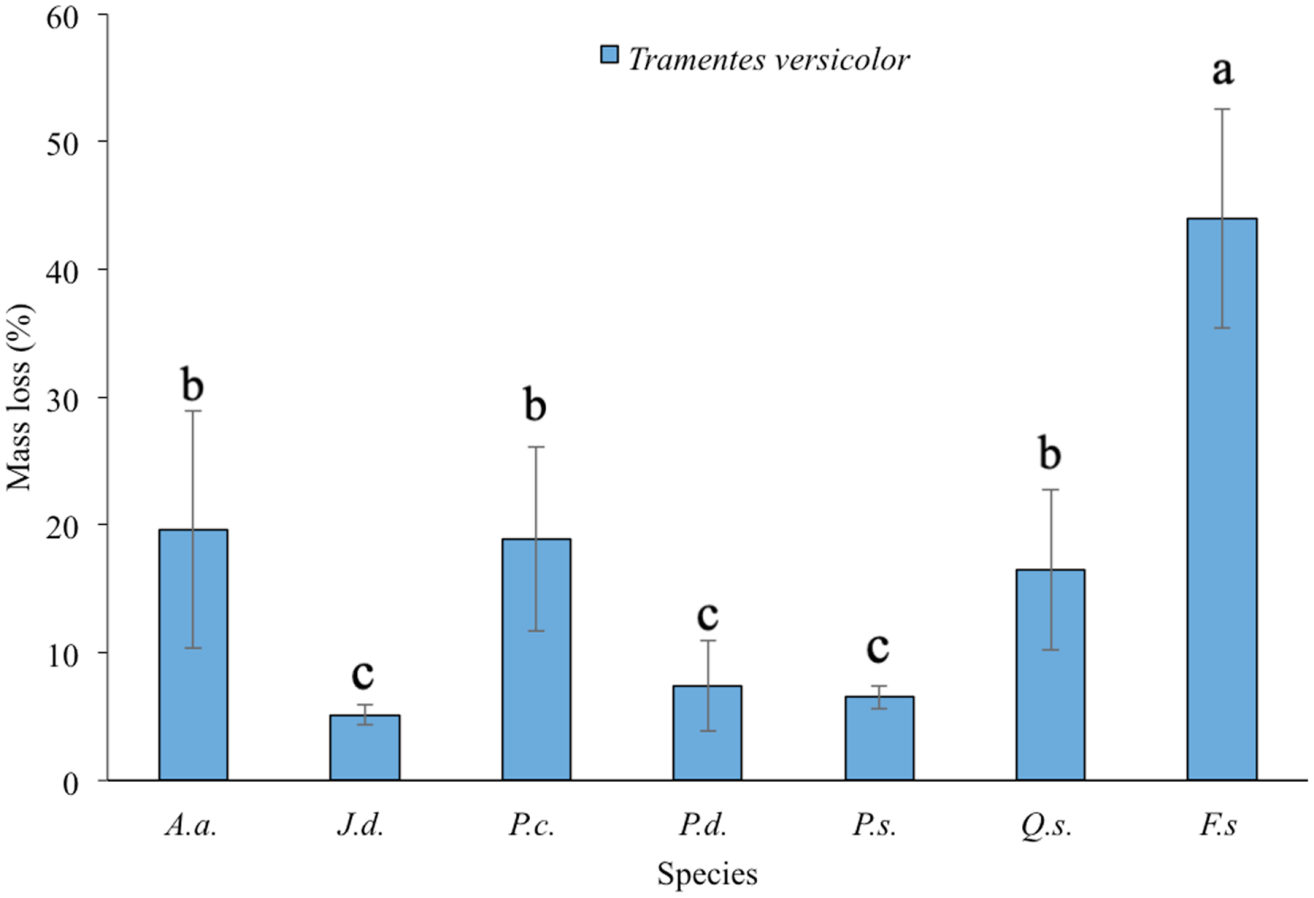
Mean ± SD of mass loss (%) of seven tree species exposed to *Trametes versicolor* in southern Durango, Mexico. Lowercase letters indicate the significant groups according to the Games–Howell test (*P* < 0.05). *J.d*. = *Juniperus deppeana*[i], P.s. The figure shows the mean ± SD of mass loss (%) of seven tree species exposed to *Trametes versicolor* in southern Durango, Mexico.

Significant differences in mass loss were observed among the tree species exposed to *Trametes versicolor* (Welch = 260.43, df = 6, 43.26; *P* < 0.0001; Table 2; Fig. 3). *Juniperus deppeana, P. strobiformis* and *P. durangensis* formed a statistical group with the lowest mass loss (around 6%), Table 1. The other three species (*Q. sideroxyla, P. cooperi* and *A. acuminata*) were similar in terms of losing a higher percentage of mass (around 18.5%), but not as much as the control *F. sylvatica* which lost 43.98% on average, thus showing that this fungal species causes greater damage in hardwood species.

**Table 2:** Descriptive statistics of mass loss (%) of seven tree species exposed to *Trametes versicolor* in southern Durango, Mexico. Values shown in this table are the mass loss of wood samples of tree species exposed to the fungus *Trametes versicolor*.

Species	*n*	Mean	SE	SD	Min	Max	X value	Durability class
*Alnus acuminata*	15	19.63	2.39	9.27	7.76	35.51	0.45	3
*Juniperus deppeana*	15	5.11	0.2	0.79	3.79	6.43	0.12	1
*Pinus cooperi*	15	18.92	1.87	7.22	4.05	25.81	0.43	3
*Pinus durangensis*	15	7.39	0.91	3.52	1.27	12.44	0.17	2
*Pinus strobiformis*	15	6.52	0.24	0.92	5.06	8.05	0.15	2
*Quercus sideroxyla*	15	16.47	1.61	6.25	5.58	26.2	0.37	3
*Fagus sylvatica*	18	43.98	2.02	8.56	36.7	62.43	1.00	N/A

**Notes:**

X value: calculated from the average mass loss of each wood species divided by the average mass loss of *F. sylvatica* after 16 weeks of fungal incubation.

Durability class: classification system according to mass loss criteria and EN 350-1. 1 = Very durable, 2 = Durable, 3 = Moderately durable, 4 = Slightly durable and 5 = Not durable.

N/A, Not available.

*n*, number of samples; SE, standard error; SD, standard deviation.

### Differences in mass loss among timber species for each fungus

**Figure 4:**
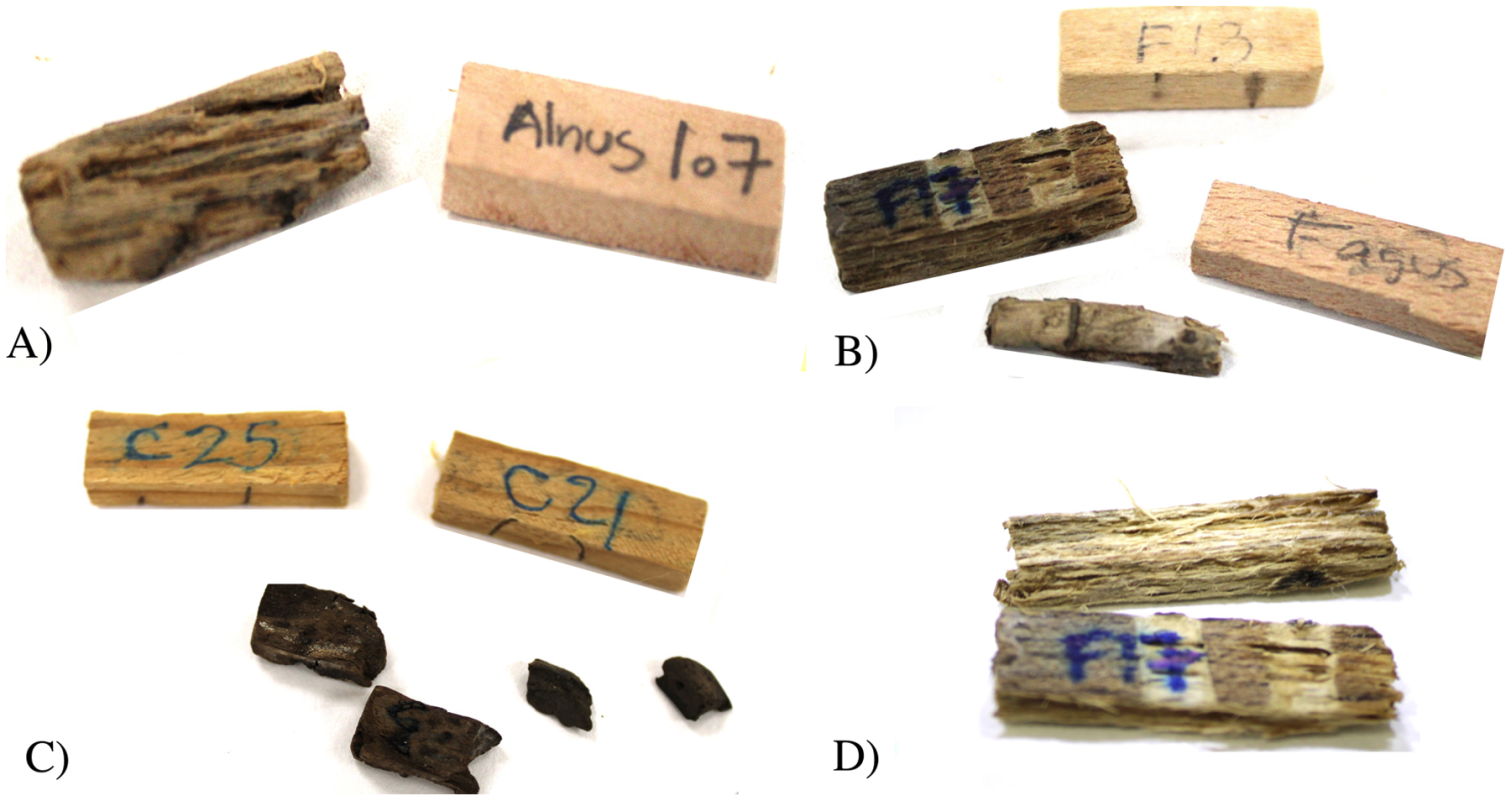
Deterioration in the wood samples analyzed. (A) *Alnus acuminata*, (B) *Fagus silvatica*, (C) *P. cooperi*, and (D) *F. sylvatica*. Photographs show the degree of deterioration in three species used.

The deterioration caused by fungi *C. puteana* and *T. versicolor* was high; some sections of the timber species *A*. *acuminata*, *P*. *cooperi*, and the control *F*. *sylvatica* were totally degraded (Fig. 4). According to the Welch t-test, *Pinus strobiformis* and *Q. sideroxyla* were significantly more susceptible to *C. puteana* damage; in contrast, *P. durangensis*, *P. cooperi* and the control (*F. sylvatica*) showed more damage due to *T. versicolor* (Table 3). *Juniperus deppeana* and *A. acuminata* showed a non-significant difference between fungal degradation effects (Table 3).

**Table 3:** Welch t-tests comparing mass loss produced by *Conidiophora puteana* and *Trametes versicolor* in each tree species in southern Durango, Mexico.

Tree species	t-Welch	df	*P*	Fungus producing more mass loss
*Alnus acuminata*	−0.268	27.806	0.791	Both fungi
*Juniperus deppeana*	0.799	27.974	0.431	Both fungi
*Pinus cooperi*	4.901	22.189	0	*T. versicolor*
*Pinus durangensis*	4.862	24.92	0	*T. versicolor*
*Pinus strobiformis*	−4.705	26.005	0	*C. puteana*
*Quercus sideroxyla*	−2.95	16.07	0.009	*C. puteana*
*Fagus sylvatica*	8.949	31.394	0	*T. versicolor*

## Discussion

Both species of fungi caused different levels of deterioration in the wood samples analyzed, as can be seen in Fig. 4 with *A. acuminata*, *P. cooperi*, and the control species *F. sylvatica*.

### Differences in mass loss among timber species for each fungus

The timber species analyzed in this study showed statistically significant levels of durability, determined by the mass loss caused by the attack of the wood-degrading fungi *Coniophora puteana* and *Trametes versicolor* (Figs. 2 and 3). The results of this work are validated according to the mass loss of *Fagus sylvatica* used as the control, which was greater than 20% for both wood-degrading fungi; this value was established by European standard 350-1 (CEN EN 350-1, 1994). No *P. sylvestris* wood samples were used to validate the deterioration in softwood species; however, *P. durangensis, P. strobiformis* and *P. cooperi* species showed loss values from 2.65% to 18.92%. In general, these three species and *J. deppeana* showed from medium to higher resistance to deterioration due to the action of fungi compared to *Q. sideroxyla* and *A. acuminata*, which were the most susceptible species to these xylophagous fungi. Similar results to those obtained in this research for *Q. syderoxila* were presented by Ayata, Akcay & Esteves (2017) in an experiment where thermal treatments were used to increase the durability of *Quercus petreae*; they found mass losses of 21.54 and 11.20% by exposing this timber species to *Pleurotus ostreatus* and *Coniophora puteana* fungi, respectively. On the other hand, the mass loss caused by *C. puteana* in *A. acuminata* in this trial was higher than the 14.66% reported by Erazo et al. (2019); however, the values were lower than the 61.84% reported by the same authors when the wood was exposed to *T. versicolor*.

### Classification of natural durability

When using the durability classification established by European standard EN 350-1 (CEN EN 350-1, 1994), it was found that *Q. syderoxyla* was not durable (Class 5) when in contact with *C. puteana*, while *A. acuminata* was slightly durable (Class 4), *J. deppeana*, *P. cooperi* and *P. strobiformis* were moderately durable (Class 3), and *P. durangensis* was very durable (Class 1). Using the same classification system, but for *T. versicolor* as a deterioration agent, *A. accuminata*, *P. cooperi* and *Q. sideroxyla* were classified as moderately durable (Class 3), while *P. durangensis* and *P. strobiformis* were durable (Class 2) and *J. deppeana* was very durable (Class 1). The low mass loss (Classes 3 and 1 when exposed to *C. puteana* and *T. versicolor*, respectively) of *J. deppeana* wood could be the result of a high content of extracts (Kirker, Bishell & Lebow, 2016). These non-structural wood components are often concentrated in different proportions in a radial direction and along the trunk of the tree, thereby avoiding biological attacks as established by Erazo et al. (2019). In Oregon, USA, *Juniperus* wood samples have shown many years of durability (>50), which is attributed to their extracts that provide a high inhibitory effect on the growth of *Gloeophyllum trabeum* and *T. versicolor* (Mun & Lynn, 2011). In contrast, several tree species produce non-toxic extracts that do not reduce damage by xylophagous fungi (de la Cruz Carrera et al., 2018). Regarding the timber species *Alnus acuminata* and *Q. sideroxyla* (Classes 4 and 5 when exposed to *C. puteana* and Class 5 when exposed to *T. versicolor*, respectively), their resistance ranged from slightly durable to not durable, with the composition of their extracts possibly being the cause. Similar studies conducted in Mexico found that *T. versicolor* caused a greater loss of mass (30.7%) in samples of *Arbutus spp*. and *Q. sideroxyla*; on the other hand, *J. deppeana, P. strobiformis, P. durangensis, P. teocote* and *P. cooperi* showed low mass losses (0.97%), placing these woods in Class 1, which corresponds to very durable (de la Cruz Carrera et al., 2018). The natural durability of the Mexican timber species tested in the present experiment was within or above the reported values for the yellow pine, *Quercus petreae*, *Pinus caribaea and Eucalyptus saligna* species (Fojutowski et al., 2014; Lana & Bernardes, 2019), which may give them an opportunity to be marketed in other countries where natural durability is an important selection factor.

## Conclusions

It is concluded that *J. deppeana, P. durangensis, P. strobiformis* and *P. cooperi* can be used in southern Durango, Mexico, in conditions of exposure to xylophagous fungi, in direct contact with the soil. However, in Mexico there is a need for more information and a better understanding about the process of microbial colonization and succession of wood. Also, the interactions among different microorganisms involved in the decay process are largely unknown and therefore further research is needed.

## Supplemental Information

10.7717/peerj.14541/supp-1 Supplemental Information 1 The mass loss of wooden samples after exposure to fungi *C. puteana* and T. versocolor.

## Additional Information and Declarations

### Data Availability

The following information was supplied regarding data availability: The raw data are available in the Supplemental File.
